# Serotonin Deficiency Rescues Lactation on Day 1 in Mice Fed a High Fat Diet

**DOI:** 10.1371/journal.pone.0162432

**Published:** 2016-09-07

**Authors:** Samantha R. Weaver, Justin C. Bohrer, Allan S. Prichard, Paola K. Perez, Liana J. Streckenbach, Jake M. Olson, Mark E. Cook, Laura L. Hernandez

**Affiliations:** 1 Department of Dairy Science, University of Wisconsin-Madison, Madison, WI, United States of America; 2 Department of Obstetrics and Gynecology, University of Wisconsin-Madison, Madison, WI, United States of America; 3 Department of Animal Sciences, University of Wisconsin-Madison, Madison WI, United States of America; University of Southampton, UNITED KINGDOM

## Abstract

Obesity is an inflammatory state associated with delayed lactogenesis stage II and altered mammary gland morphology. Serotonin mediates inflammation and mammary gland involution. The objective of this study was to determine if a genetic deficiency of tryptophan hydroxylase 1, the rate-limiting enzyme in peripheral serotonin synthesis, would result in an improved ability to lactate in dams fed a high fat diet. Twenty-six female mice were fed a high (HFD) or low fat (LFD) diet throughout pregnancy and lactation. Fourteen mice were genetically deficient for *Tph1* (*Tph1*^*-/-*^), and twelve were wild type. Milk yield, pup mortality, and dam weights were recorded and milk samples were collected. On day 10 of lactation, dams were sacrificed and mammary glands were harvested for RT-PCR and histological evaluation. HFD dams weighed more than LFD dams at the onset of lactation. WT HFD dams were unable to lactate on day 1 of lactation and exhibited increased pup mortality relative to all other treatments, including *Tph1*^*-/-*^ HFD dams. mRNA expression of immune markers C-X-C motif chemokine 5 and tumor necrosis factor alpha were elevated in WT HFD mammary glands. Mammary gland histology showed a reduced number of alveoli in WT compared to *Tph1*^*-/-*^ dams, regardless of diet, and the alveoli of HFD dams were smaller than those of LFD dams. Finally, fatty acid profile in milk was dynamic in both early and peak lactation, with reduced de novo synthesis of fatty acids on day 10 of lactation in the HFD groups. Administration of a HFD to C57BL/6 dams produced an obese phenotype in the mammary gland, which was alleviated by a genetic deficiency of *Tph1*. Serotonin may modulate the effects of obesity on the mammary gland, potentially contributing to the delayed onset of lactogenesis seen in obese women.

## Introduction

Breastfeeding provides significant health benefits to both infant and mother. Longer durations of breastfeeding have been associated with reduced cholesterol and blood pressure, along with reduced incidence of type 2 diabetes in the mother. Breastfed infants have been demonstrated to show improved cognitive development, are likely protected against immune-related diseases, and have a reduced risk of obesity [1,2]. The American Academy of Pediatrics recommends exclusive breastfeeding for six months, followed by continued breastfeeding for one year or longer [3]. An important barrier to exclusive breastfeeding is delayed onset of lactogenesis stage II (DOL), defined as the onset of copious milk secretion. Difficulties with breastfeeding during the first week postpartum are associated with a greater risk of early termination of breastfeeding, as well as a lower chance of breastfeeding subsequent children [4]. There is a well-established relationship between maternal body mass index and DOL, with overweight and obese women more likely to experience DOL and terminate breastfeeding early [4,5,6,7]. In the United States, approximately one third of women of childbearing age are overweight or obese [8,9]. While various mechanical theories have been proposed to explain the correlation between DOL and obesity, including sore nipples from poor latching of the infant due to difficulty positioning, little effort has focused on the physiological or molecular mechanisms driving DOL in obese women [10,11]. Although it has been shown that women who were overweight or obese had lower prolactin response to suckling than normal-weight women at 48 hours and 7 days post-partum, local mammary gland dynamics in response to obesity have yet to be explored [12].

Obesity is characterized as a state of inflammation. Overweight and obese individuals have various elevated circulating cytokines [2] and, in the obese rodent mammary gland, necrotic adipocytes are surrounded by macrophages to form crown-like structures which are associated with proinflammatory mediators tumor necrosis factor alpha (*Tnfa*) and interleukin 1 beta (*Il1b*) [13]. Inflammatory pathways are also implicated in mammary gland involution, or the cessation of lactation. Macrophages and monocytes are heavily recruited to the involuting mammary gland, and there is a marked increase in pro-inflammatory cytokines within 12 hours of involution [14,15]. Involution is known to be mediated in a dose-dependent manner in the mammary gland by peripheral serotonin (5-hydroxytryptamine; 5-HT) [16,17].

Serotonin is synthesized in non-neuronal tissues in a two-step process from the amino acid L-tryptophan. L-tryptophan is converted to 5-hydroxytryptophan (5-HTP) by the rate-limiting enzyme tryptophan hydroxylase (TPH) and 5-HTP is further decarboxylated to form 5-HT [18]. There are two isoforms of TPH; the neuronal isoform TPH2 catalyzes the formation of 5-HT in the CNS, while non-neuronal 5-HT is synthesized via TPH1. Mice injected with 5-HT have inhibited weight gain when fed a high fat diet (HFD) compared to mice fed standard chow [19] and inhibition of peripheral 5-HT was shown to reduce obesity through elevation of brown adipose tissue activity [20]. In addition, peripheral 5-HT is a potent immune modulator and 5-HT receptors are present on a variety of immune cell types [21]. Work by Hernandez et al. (2012) demonstrated that administration of a HFD to lactating rats increased the mRNA expression of *Tph1* in the mammary gland, along with elevated levels of inflammatory marker *Tnfa* [22]. Finally, obese patients have demonstrated reduced circulating serotonin concentrations relative to non-obese patients, potentially resulting in reduced lipolysis and increased lipid storage [23]. Additionally, numerous pharmacotherapies used in the treatment of obesity manipulate both the central and peripheral serotonin systems [24]. As such, use of mice deficient for *Tph1* may mimic phenotypes of obesity in human patients. Given the numerous correlations between 5-HT, maintenance of lactation, and obesity, the objective of this study was to determine if a genetic deficiency for *Tph1* would improve lactation outcomes in combination with HFD administration. We hypothesized that *Tph1*-deficient lactating dams fed a HFD would have an improved ability to lactate when compared to their wild type counterparts. Additionally, we anticipated an increased immune response in wild type HFD dams that would be attenuated by *Tph1* deficiency. Through these objectives, we hoped to establish a molecular basis for delayed onset of lactation in obese women, implicating the serotonergic system.

## Methods

### Animal Handling and Diets

All experiments were performed under protocols approved by the Research Animal Care and Use Committee at the University of Wisconsin-Madison. Female mice on the C57BL/6 background were individually housed in a controlled environmental facility for biological research in the Animal Science Department at the University of Wisconsin-Madison. All mice were obtained through our mating colony in which we maintain *Tph1*^*-/-*^ mice on the C57BL/6 background [25, 26]. Mice were maintained at a temperature of 25°C and humidity of 50%–60% on a 12-h light/dark cycle with free access to food and water. At 5 weeks of age, mice were enrolled on a purified diet containing either 10% kcal% fat (Research Diets, New Brunswick, NJ No. D12450B) or 60% kcal% fat (Research Diets, New Brunswick, NJ No. D12492) (Table 1). Feed intake was measured twice per week throughout the course of the study. Following a loading period of 3 weeks on the diet, mice were initially bred overnight with a male at 8 weeks of age. If mice did not become pregnant and maintain the pregnancy by their second pregnancy, they were removed from the study. Dams were assigned to one of four treatments: wild-type (WT) dams fed a LFD (WT LFD; n = 8), WT dams fed a HFD (WT HFD; n = 4), *Tph1* deficient dams fed a LFD (*Tph1*^*-/-*^ LFD; n = 8) and *Tph1* deficient dams fed a HFD (*Tph1*^*-/-*^HFD; n = 6). Given the difficulty of achieving and maintaining pregnancy in the HFD groups, litters were not standardized. Sex differences were not recorded and males and female pups were pooled for all offspring measurements.

**Table 1:** Composition of Control and High Fat Diets^a^.

Diet Composition	Control Diet (D12450B)	High Fat Diet (D12492)
**Macronutrients**		
Protein, *%kcal*	20	20
Carbohydrate, *%kcal*	70	20
Fat, *%kcal*	10	60
Total, *%kcal*	100	100
Energy, *kcal/g*	3.85	5.24
**Ingredients, *g***		
Casein, 30 Mesh (D12450B) or 80 mesh (D12492)	200	200
L-Cysteine	3	3
Corn Starch	315	0
Maltodextrin 10	35	125
Sucrose	350	68.8
Cellulose, BW200	50	50
Soybean Oil	25	25
Lard^b^	20	245
Mineral Mix S10026	10	10
DiCalcium Phosphate	13	13
Calcium Carbonate	5.5	5.5
Potassium Citrate, 1 H2O	16.5	16.5
Vitamin Mix V10001	10	10
Choline Bitartrate	2	2
FD&C Yellow Dye #5 (D12450B), FD%C Blue Dye #1 (D12492)	0.05	0.05

^a^Diets were formulated and purified by Research Diets (www.researchdiets.com).

^b^Typical analysis of cholesterol in lard = 0.72 mg/g.

### Sample Collection

The number of pregnancies were recorded for each dam. Many dams resorbed their litters, as well as killed all their pups at birth, often requiring numerous matings to achieve lactation. Dam weights were measured before enrollment on the diet, on day 7 of pregnancies (P7), between day 17 to 20 of pregnancy (P17-20), on day 0 of lactation (L0), and on day 10 of lactation (L10). Pup mortality was measured daily throughout lactation. Milk yield was determined daily throughout lactation using the weigh-suckle-weigh (WSW) method. Briefly, pups were removed from their mothers at 0800 h. After being separated for 4 h, each litter was weighed and then at 1200 h returned to their mothers to nurse for 45 min. After 45 minutes of suckling, each litter was weighed again to estimate milk yield. Milk yields were standardized per pup by dividing the total milk yield for the litter by the number of pups in each litter on each day of lactation [22]. Milk was collected in the first few days post-partum (the first day of lactation (L1) for all groups except WT HFD, which was collected on day 2–3 of lactation) and on L10. To obtain milk, dams were anesthetized with isofluorane and intramuscularly injected with 0.6 U of purified oxytocin (Agrilabs) to stimulate milk ejection. On L10, dams were euthanized via CO_2_ asphyxiation and mammary gland number 4 was collected for RNA and total protein isolation. Tissue was flash frozen in liquid nitrogen and stored at −80°C until processed. The opposing number 4 mammary gland was fixed in 4% paraformaldehyde overnight at 4°C and then embedded in paraffin and sectioned (5 μm) for histological evaluation through hematoxylin and eosin staining.

### Mammary gland RNA extraction and Quantitative Real-time PCR

Total RNA was extracted from mammary gland tissue using TRI-Reagent (Molecular Research) and was reverse transcribed (1 μg) to cDNA using Bio-Rad iScript Reverse Transcription Supermix (#1708840). Quantitative RT-PCR was conducted with the CFX96 Touch Real-Time PCR Detection System (Bio-Rad). Reaction mixtures and cycling conditions were performed as previously described [27]. All primers were designed to span exon-exon junctions and for an optimal annealing temperature of 60°C. Amplification efficiencies of primers were accepted within a range of 95 to 105% efficiency and primer specificity was assessed by the presence of a single temperature dissociation peak, eliminating any primers with indication of secondary structures. Primer sequences can be found in Table 2. The geometric mean of *β-Actin* (*Actb*), *β-2-microglobulin (B2m)*, and *Heat shock protein 90kDa Alpha Family Class B Member 1 (Hsp90ab1)* was calculated and used as the housekeeping parameter, and analysis was conducted using the 2^−ΔΔCt^ method [28].

**Table 2:** Primer Sequences for Genes Quantified by Real-Time PCR^a^.

Gene	GeneBank #	Sequence
*Actb*	NM_007393	Forward 5’-TACAGCTTCACCACCACAGC-3’ Reverse 3’-CTTCTCCAGGGAGGAAGAGG-5’
*B2m*	NM_009735	Forward 5’-TGGTGCTTGTCTCACTGACC-3’ Reverse 3’-CGGGTGGAACTGTGTTACG-5’
*Hsp90ab1*	NM_008302	Forward 5’-ACTGCTCTGCTCTCCTCTGG-3’ Reverse 3’-GGGATCTCATCAGGAACAGC-5’
*Cck*	NM_031161	Forward 5’-ACTGCTAGCGCGATACATCC-3’ Reverse 3’-CCCACTACGATGGGTATTCG-5’
*Cxcl5*	NM_009141	Forward 5’-TCGTGTTTGTCACTCGAAGG-3’ Reverse 3’-GGGATTACTGAGTGGCATCC-5’
*Igfbp5*	NM_010518	Forward 5’-CCTTGAGTGTGCCTCTGTCC-3’ Reverse 3’-ACAAGTTTGGGGGAGGTAGG-5’
*Nos2*	NM_010927	Forward 5’-GTGGTGACAAGCACATTTGG-3’ Reverse 3’-AAGGCCAAACACAGCATACC-5’
*Pfkfb3*	NM_001177752	Forward 5’-CAGCTACCAGCCTCTTGACC-3’ Reverse 3’-TGTACTCATTCTCGCCATGC-5’
*Tnfa*	NM_013693	Forward 5’-AAAGGGGATTATGGCTCAGG-3’ Reverse 3’-CTCCCTTTGCAGAACTCAGG-5’
*Tph1*	NM_009414	Forward 5’-TTCACCATGATTGAAGACAAC-3’ Reverse 3’-TCCGACTTCATTCTCCAAGG-5’

^a^All primers were designed using the Primer3 Input v.0.4.0. (http://bioinfo.ut.ee/primer3-0.4.0/).

GeneBank accession numbers are listed beside the primer name. Primer sequences are presented as 5’ to 3’ (forward) and 3’ to 5’ (reverse). *Actb*—β-actin; *B2m*—β-2-microglobulin; *Hsp90ab1*—Heat shock protein 90kDa Alpha Family Class B Member 1; *Cck*—cholecystokinin; *Cxcl5* -chemokine (C-X-C motif) ligand 5; *Igfbp5*—insulin-like growth factor binding protein 5; *Nos2*—nitric oxide synthase 2; *Pfkfb3*—6-phosphofructo-2-kinase/fructose-2,6-bisphosphatase 3; *Tnfa*—tumor necrosis factor α; *Tph1*—tryptophan hydroxylase 1.

### Mammary Gland Protein Isolation, Protein Assays, and Histology

Protein was isolated from L10 mammary gland tissue using radioimmunoprecipitation buffer (RIP) plus 10 μL/mL of Halt Protease and Phosphatase Inhibitors Cocktail (Thermo Scientific #78441). Protein concentrations were determined using the bicinchoninic acid assay (Pierce Chemicals #23227).

Mammary gland concentration of 5-HT was determined following the manufacturer’s instructions using a Serotonin EIA Kit (IM1749, Immunotech, Beckman Coulter), loading 50 μg of protein per sample. The intra-assay CV was 1.7%. Mammary gland concentration of TNFa was evaluated using an ELISA following the manufacturer’s instructions, loading 50 μg of protein per sample (Thermo Scientific KMC3011). The intra-assay CV was 3.2%.

On L10, the number 4 mammary gland was removed and fixed in 4% paraformaldehyde overnight at 4°C. It was then transferred to 70% ethanol until dehydration with xylene and paraffin embedding. Paraffin blocks were sectioned at 5 μm. For histological visualization, sectioned mammary glands were stained with hematoxylin and eosin (H&E). Two sections were stained from each dam. Three images were taken of each section (resulting in 6 images per dam) at a 20x objective. Using ImageJ software (NIH Version 1.49), alveoli number and diameter were quantified. Only alveoli whose borders were completely within the field of the image were counted and measured. All data were averaged across all images.

### Milk Fatty Acid Profile

Total fat of milk samples was extracted with Folch reagent as previously described [29]. Bound fatty acids were methylated using 0.5M sodium methoxide similar to methods described by Christie (1982) using select modifications described by Politz et al., (2013) [30,31]. Briefly, toluene was added to dried chloroform extract (2:1 v/w). Next, 0.5M sodium methoxide was added in excess to lipid extracts (100:1 v/w) and samples were heated at 60°C for 10 minutes in a water bath. The methylation reaction was quenched with 0.35M glacial acetic acid (1.5:1 v/v) followed by hexane extraction of methyl esters to yield a final FAME concentration of 10mg/ml. Relative abundance of fatty acid methyl esters (FAME) was analyzed using gas chromatography (Agilent 6890N) coupled with flame ionization detection as previously described [32]. A 100m biscyanopropyl polysiloxane capillary column (Rt-2560, Restek Corp, Bellefonte, PA) was used for separation of FAMEs.

### Statistical Analysis

Statistical analysis was performed using Prism version 6.0h (GraphPad Software). Gene and protein expression, alveoli size and number, and pup mortality were analyzed using a two-way ANOVA followed by Tukey’s post hoc test for differences between groups. Outliers were determined and removed as necessary. Dam body weight, milk yield, and milk fatty acid profile data was analyzed using two-way ANOVA with treatment, time, and the interaction between treatment and time as main effects. Multiple comparisons were made using the Holm-Sidak method to detect differences between treatment groups across time points. Outliers were identified using the ROUT method. Differences between means were considered significant at P<0.05. All values are reported as means ± SEM.

## Results

### Dam weight and pup mortality are elevated in WT HFD relative to *Tph1*^-/-^ HFD mice

Both of the HFD groups had a numerically higher number of pregnancies than their LFD counterparts (1.5 ± 0.3 versus 1.7 ± 0.2 versus 1.4 ± 0.2 versus 1.3 ± 0.2 pregnancies in WT HFD, *Tph1*^-/-^ HFD, WT LFD, and *Tph1*^-/-^ LFD, respectively), although this was not significant (Table 3). Only 2 out of 6 *Tph1*^-/-^ HFD dams and 2 out of 4 WT HFD dams (compared to 5 out of 8 WT LFD dams and 7 out of 10 *Tph1*^-/-^ LFD dams) carried their litters through pregnancy and fed their pups through L10 on their first mating. The remaining dams included in the study required an additional mating to achieve a successful lactation (Table 3). It also took both of the HFD groups a numerically, but not significantly, greater number of days from the initial breeding to produce a litter that survived through L10 (31.3 ± 5.7 versus 27.0 ± 3.3 versus 24.6 ± 2.3 versus 24.0 ± 2.3 days in WT HFD, *Tph1*^-/-^ HFD, *Tph1*^-/-^ LFD, and WT LFD, respectively) (Table 3).

**Table 3:** Dam Pregnancy and Litter Mortality Outcomes.

	WT LFD	WT HFD	*Tph1*^-/-^ LFD	*Tph1*^-/-^ HFD
Average Number of Pregnancies	1.4 ± 0.2	1.5 ± 0.3	1.3 ± 0.2	1.7 ± 0.2
Number of Dams with a Successful First Pregnancy and Lactation (%)	5 / 8 (63%)	2 / 4 (50%)	7 / 10 (70%)	2 / 6 (33%)
Days from First Mating to Birth of a Litter that Survived through L10	24.0 ± 2.3	31.3 ± 5.7	24.6 ± 2.3	27.0 ± 3.3
Litter Size on L0 (pups)	7.0 ± 0.4	7.0 ± 0.7	7.3 ± 0.7	7.5 ± 0.7
Litter Size on L1 (pups)	6.9 ± 0.5	4.0 ± 0.7	6.0 ± 0.8	5.8 ± 0.5
Pups Dead from L0 to L1 (% of total pups from all litters)	2.1 ± 2.1	43 ± 6.9	12 ± 4.1	20 ± 7.7
Litter Size on L10 (pups)	6.4 ± 0.8	3.3 ± 0.6	5.6 ± 0.8	5.3 ± 0.7

**Fig 1:**
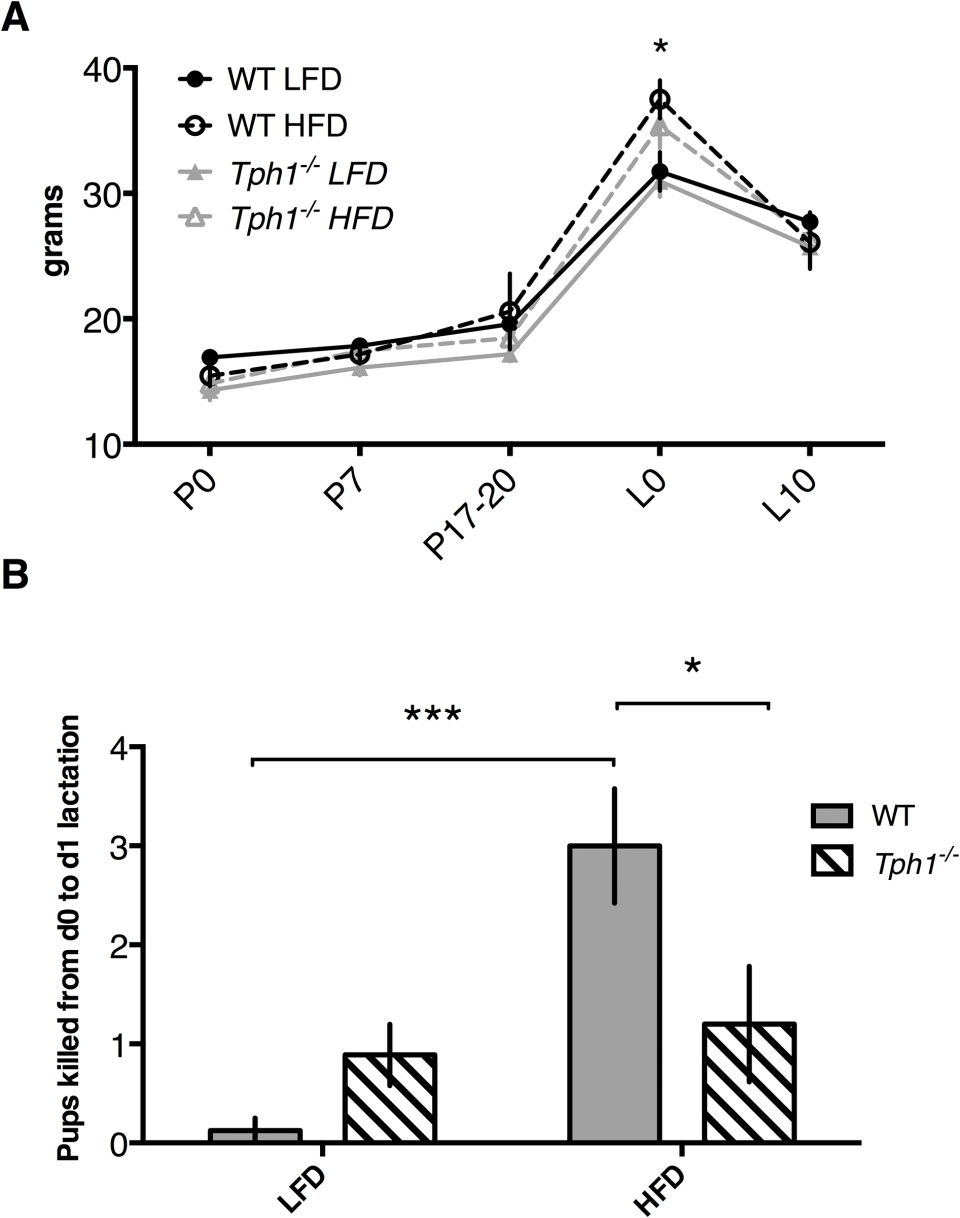
All dams fed a high fat diet weighed more than those fed a low fat diet, but only wild type high fat diet fed dams killed more pups at the onset of lactation. Wild type or *Tph1* deficient dams were either fed a high fat or low fat diet throughout pregnancy and lactation. Shown in (*A*) are the effects on dam weight and in (*B*) on pup mortality. Values are means ± SEMs. Stars indicate statistical significance between groups (* = 0.05<*P*<0.01, ** = 0.01<*P*<0.001, *** = 0.001<*P*<0.0001). WT, wild type; *Tph1*^*-/-*^, *Tph1* deficient; LFD, low fat diet; HFD, high fat diet; P0, day 0 of pregnancy; P7, day 7 of pregnancy; P17-20, day 17 to 20 of pregnancy; L0, day 0 of lactation; L10, day 10 of lactation.

To evaluate if dams were responding to the diet, we measured dam body weight at several time points throughout the experiment. There was an overall effect of treatment (*P* = 0.001), time (*P*<0.0001), and a tendency towards the interaction of treatment and time (*P* = 0.09) with respect to dam body weight (Fig 1A). All dams reached peak body weight on L0, with *Tph1*^*-/-*^HFD weighing more than *Tph1*^*-/-*^ LFD (*P*<0.05; 35.4 ± 1.8 versus 31.0 ± 1.2 grams for *Tph1*^*-/-*^ HFD and *Tph1*^*-/-*^ LFD, respectively). Additionally, on L0, WT HFD dams weighed more than WT LFD dams (*P*<0.01; 37.5 ± 1.5 versus 31.8 ± 1.5 grams for WT HFD and WT LFD, respectively).

Given our hypothesis that serotonin mediates an involution phenotype, it was necessary to evaluate if dams were able to supply enough milk for their pups to survive. To this end, we measured pup mortality at the onset of lactation (L0 to L1) to mimic DOL in women. Diet had an effect on pup mortality (*P* = 0.0004), as did the interaction of diet and genotype (*P* = 0.0003) (Fig 1B). WT HFD dams had more dead pups than WT LFD dams (*P*<0.001; 3.0 ± 0.6 versus 0.13 ± 0.13 for WT HFD and WT LFD, respectively). Ablation of *Tph1* attenuated the effects of the HFD, with *Tph1*^*-/-*^ HFD dams having less dead pups than WT HFD dams (*P*<0.05; 1.2 ± 0.6 pups dead for *Tph1*^*-/-*^ HFD). Genotype alone did not affect pup mortality (*P*>0.05), with 0.89 ± 0.31 dead pups from *Tph1*^*-/-*^ LFD dams between L0 and L1. Average litter sizes as well as average mortality rates among treatments are presented in Table 3.

### Immune and serotonergic mRNA expression is elevated in WT HFD dams

**Fig 2:**
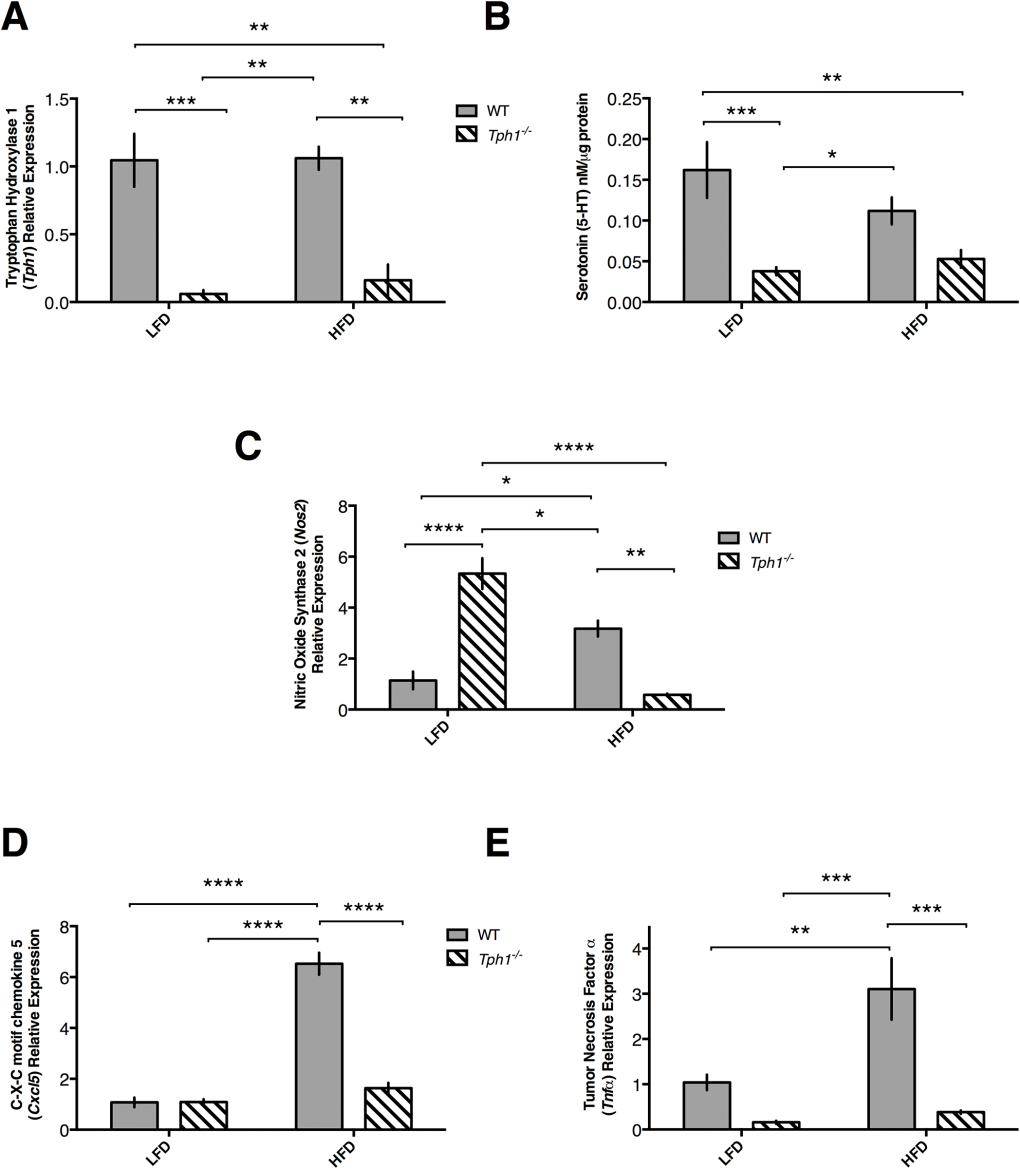
Mammary gland mRNA and protein expression of various serotonergic and immune markers is altered in response to high fat diet and *Tph1* deficiency. Wild type or *Tph1* deficient dams were either fed a high fat or low fat diet throughout pregnancy and lactation. Shown is (*A*) mammary gland mRNA expression of tryptophan hydroxylase 1, (*B*) total mammary gland protein expression of tryptophan hydroxylase 1, (*C*) mammary gland mRNA expression of nitric oxide synthase, (*D*) mammary gland mRNA expression of C-X-C motif chemokine 5, (*E*) mammary gland mRNA expression of tumor necrosis factor alpha. Values are means ± SEMs. Stars indicate statistical significance between groups (* = 0.05<*P*<0.01, ** = 0.01<*P*<0.001, *** = 0.001<*P*<0.0001, **** = *P*<0.0001). WT, wild type; *Tph1*^*-/-*^, *Tph1* deficient; LFD, low fat diet; HFD, high fat diet.

To ensure that transcription and translation of 5-HT was reduced in the mammary glands of *Tph1*^*-/-*^ dams, we evaluated *Tph1* mRNA and subsequent 5-HT protein expression. There was no effect of diet (*P*>0.05) in either of the treatment groups. As anticipated, mRNA expression of *Tph1* was almost zero in both *Tph1*^*-/-*^ treatment group mammary glands (*P*<0.0001; Fig 2A). Additionally, while there was no effect of diet on 5-HT content in the mammary gland (*P*>0.05), but 5-HT content in WT mammary glands was higher than in both *Tph1*^*-/-*^ treatment groups’ mammary glands (*P* = 0.0002; Fig 2B). Interestingly, there tended to be an interaction between diet and genotype (P = 0.08) in 5-HT content.

We then evaluated expression of several immune markers in the mammary gland to correlate serotonergic activity with immune activity on L10. Genotype had an overall effect on nitric oxide synthase 2 (*Nos2*) mRNA expression (*P* = 0.04), as did diet (*P* = 0.003) and the interaction of genotype and diet (*P*<0.0001). Within WT dams, *Nos2* expression was elevated by HFD (*P*<0.05). Deficiency of *Tph1* decreased *Nos2* expression in HFD dams relative to WT dams (*P*<0.01). Deficiency of *Tph1* within LFD dams increased expression of *Nos2* (*P*<0.0001) and within *Tph1* deficient mice, HFD dams had reduced *Nos2* mRNA expression relative to *Tph1*^*-/-*^ LFD dams (*P*<0.0001; Fig 2C). Genotype (*P*<0.0001), diet (*P*<0.0001), and the interaction (*P*<0.0001) all had an effect on mRNA expression of chemokine (C-X-C motif) ligand 5 (*Cxcl5*). WT HFD dams had greater *Cxcl5* expression than WT LFD dams (*P*<0.0001) and *Tph1* deficiency attenuated the effect of HFD (*P*<0.0001). There was no effect of *Tph1* deficiency within LFD dams (*P*>0.05; Fig 2D). mRNA expression of *Tnfa* was overall affected by genotype (*P*<0.001), diet (*P*<0.01), and the interaction (*P*<0.05). WT HFD dams had elevated expression of *Tnfa* over WT LFD dams (P<0.01), which was attenuated by deficiency of *Tph1* in HFD dams (*P*<0.001). Similar to *Cxcl5*, diet had no effect on *Tnfa* expression within LFD dams (*P*>0.05; Fig 2E). Although not statistically significant, there was numerically higher expression of TNFa protein in the L10 WT HFD mammary glands as well (156 ± 33.8 versus 147 ± 9.6 versus 138 ± 11.4 versus 108 ± 18.0 pg/mL for WT HFD, WT LFD, *Tph1*^*-/-*^ LFD and *Tph1*^*-/-*^ HFD, respectively).

### Mammary gland alveolar dynamics are affected by both diet and serotonergic activity

**Fig 3:**
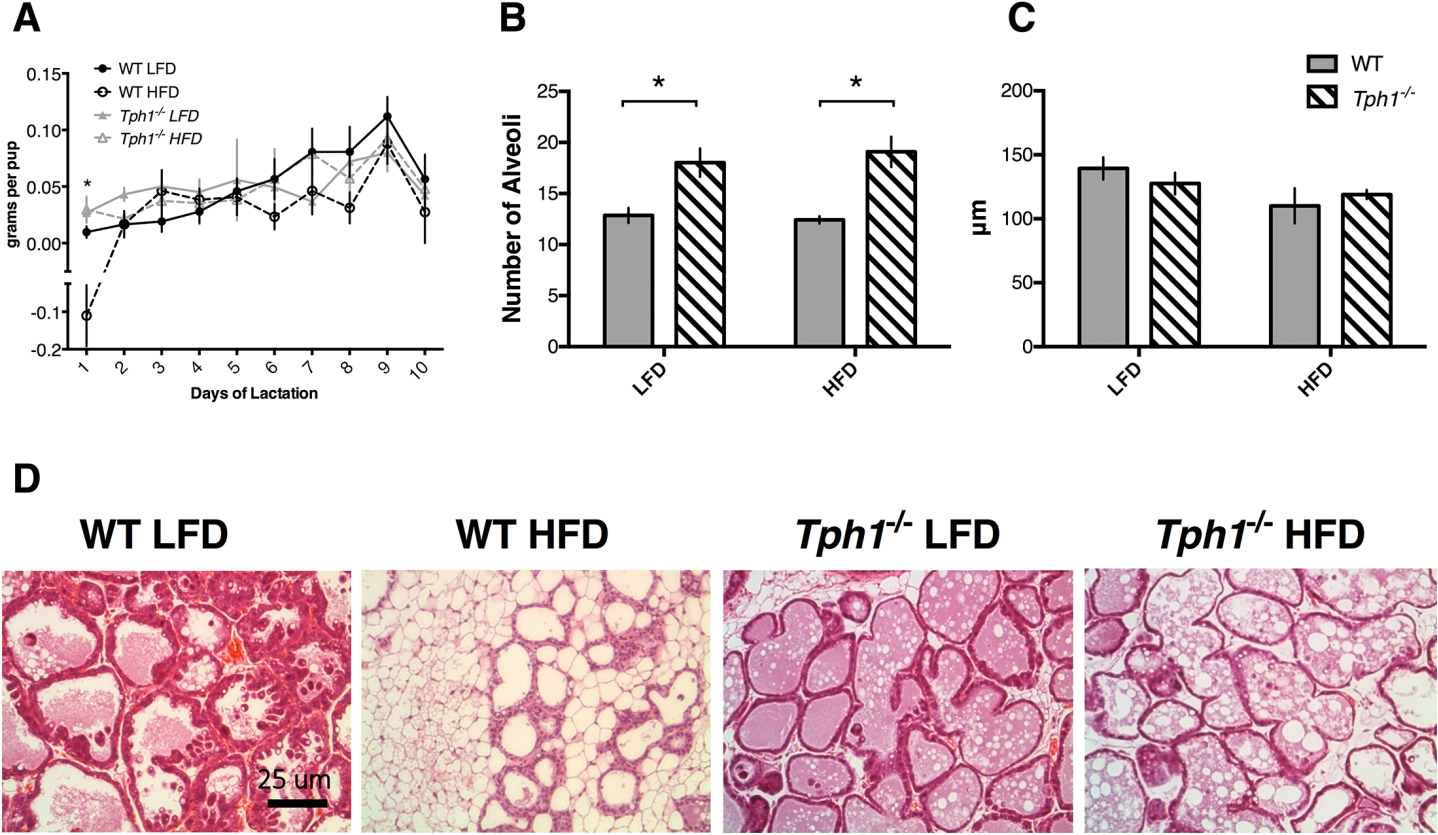
Milk yield and mammary gland morphology are altered in response to high fat or low fat diet in wild type and *Tph1* deficient dams. Wild type or *Tph1* deficient dams were either fed a high fat or low fat diet throughout pregnancy and lactation. Shown are the effects on (*A*) milk yield (grams per pup), (*B*) number of alveoli in the mammary gland, (*C*) size of alveoli in the mammary gland (μm), (*D*) mammary gland morphology as visualized by hematoxylin and eosin staining. Values are means ± SEMs. Stars indicate statistical significance between groups (* = 0.05<*P*<0.01, ** = 0.01<*P*<0.001, *** = 0.001<*P*<0.0001, **** = *P*<0.0001). WT, wild type; *Tph1*^*-/-*^, *Tph1* deficient; LFD, low fat diet; HFD, high fat diet.

In order to examine the effects of obesity and 5-HT on DOL on milk production, we measured milk yield and quantified alveolar activity. With respect to milk yield across the entire lactation, there was an effect of treatment (*P* = 0.04) and time (*P*<0.0001), but there was no interaction between treatment and time (Fig 3A). In particular, on L1, WT HFD dams were unable to produce milk compared to the other treatment groups (*P*<0.05), with pups actually losing weight across the weigh-suckle-weigh period (-0.1 ± 0.08 versus 0.01 ± 0.005 versus 0.03 ± 0.006 versus 0.03 ± 0.012 grams per pup from WT HFD, WT LFD, *Tph1*^*-/-*^ LFD and *Tph1*^*-/-*^ HFD, respectively). There was an effect of genotype on the number of alveoli in the dams’ mammary glands (*P* = 0.0004), but no overall effect of diet or the interaction of genotype and diet (*P*>0.05). Within WT dams, there was no effect of diet (*P*>0.05). However, within HFD and LFD dams, the dams deficient for *Tph1* had a reduced number of alveoli compared to WT mice (*P*<0.05; Fig 3B). There was an overall effect of diet on the diameter of alveoli (*P*<0.05). While WT HFD dams had numerically smaller alveoli than WT LFD dams, this effect was not significant. Similarly, WT HFD dams had smaller alveoli than *Tph1*^*-/-*^ HFD dams, but not significantly so (Fig 3C). Upon visualization of mammary gland H&E, significant deposits of fat were noted in the WT HFD mammary glands, with less milk in the alveoli (Fig 3D).

### Fatty acid milk and mRNA profiles are responsive to both HFD and *Tph1* ablation

**Fig 4:**
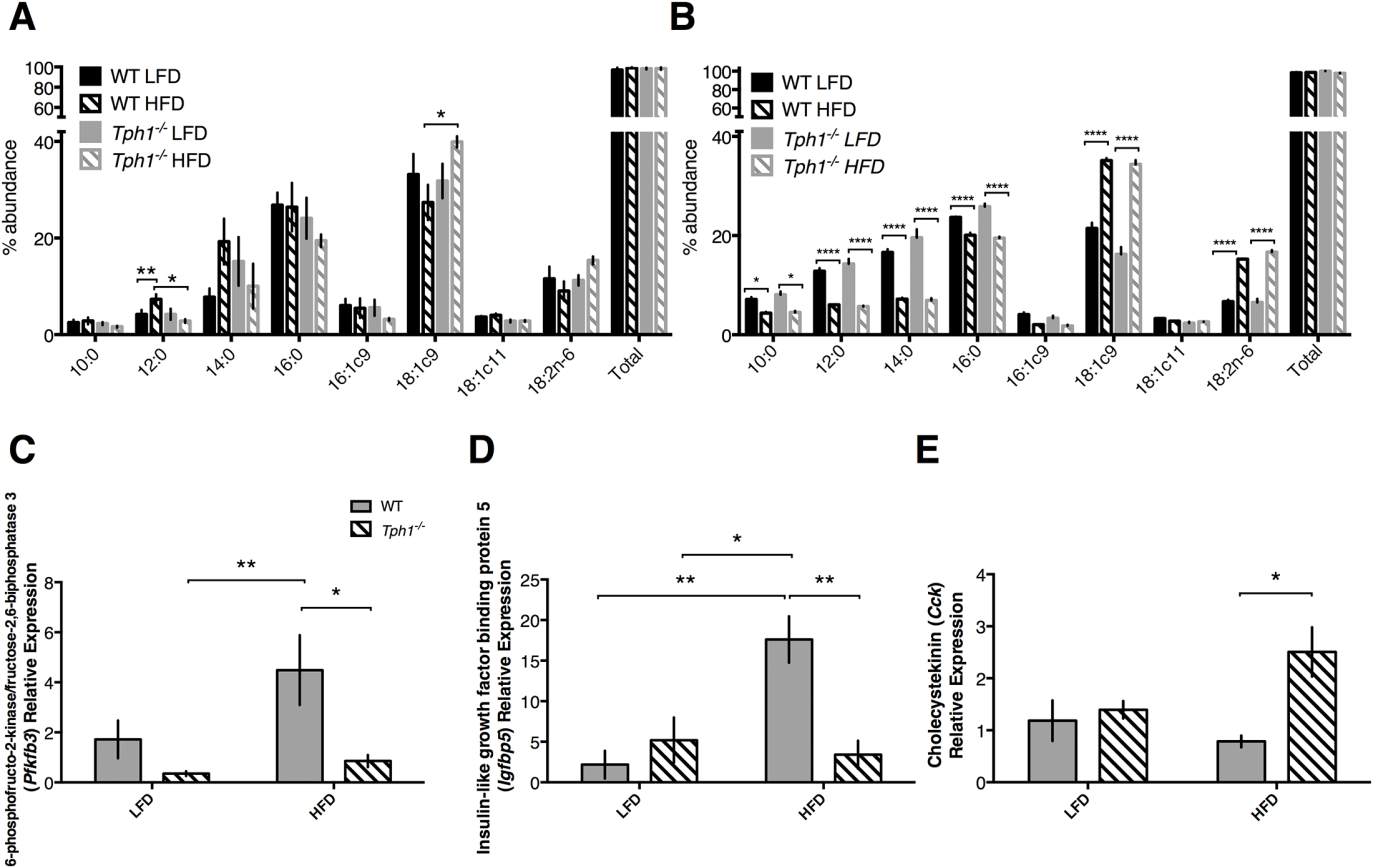
Milk fatty acid composition and mRNA expression of fat-related genes were altered in response to high fat or low fat diet fed to wild type or *Tph1* deficient dams. Wild type or *Tph1* deficient dams were either fed a high fat or low fat diet throughout pregnancy and lactation. Shown are the effects on (*A*) relative abundance of various fatty acids in milk from early lactation, (*B*) relative abundance of various fatty acids in milk on day 10 of lactation, (*C*) mammary gland mRNA expression of 6-phosphofructo-2-kinase/fructose-2,6-bisphosphatase 3, (*D*) mammary gland mRNA expression of insulin-like growth factor binding protein 5, (*E*) mammary gland mRNA expression of cholecystekinin. Values are means ± SEMs. Stars indicate statistical significance between groups (* = 0.05<*P*<0.01, ** = 0.01<*P*<0.001, *** = 0.001<*P*<0.0001, **** = *P*<0.0001). WT, wild type; *Tph1*^*-/-*^, *Tph1* deficient; LFD, low fat diet; HFD, high fat diet.

In order to establish the impact of diet and 5-HT on fatty acid profiles, we examined fatty acid abundance in milk collected on the first day possible (L1 for all treatment groups, except the WT HFD which was on day 2–3 of lactation) and on L10. In early lactation samples, there was a significant effect dependent on the length of fatty acid chain (*P*<0.0001), and there was an overall interaction with the treatment and length of fatty acid chain (*P* = 0.04; Fig 4A). Specifically, there was a decreased concentration of 12:0 fatty acids (lauric acid) in the WT LFD milk relative to the WT HFD milk (*P*<0.01). There was also decreased concentration of lauric acid in *Tph1*^*-/-*^ HFD relative to WT HFD (*P*<0.05) in the early lactation milk. Finally, on L1, there was a reduced concentration of 18:1c9 fatty acids (oleic acid) in the milk of WT HFD dams relative to *Tph1*^*-/-*^ HFD dams (*P*<0.05). On L10, there was a larger change in the fatty acid chains under 16:0 in length, with all chains decreased in concentration in the WT HFD relative to WT LFD and *Tph1*^*-/-*^ HFD relative to *Tph1*^*-/-*^ LFD (*P*<0.05 in the 10:0 versus *P*<0.0001 in all other fatty acid chains up to 16:0; Fig 4B). By contrast, the concentration of fatty acids 18:1c9 and 18:2n-6 were increased in the milk of WT LFD and *Tph1*^*-/-*^ LFD relative to WT HFD and *Tph1*^*-/-*^ HFD, respectively (*P*<0.0001).

The mRNA expression of various fatty acid pathway-associated genes such as 6-phosphofructo-2-kinase/fructose-2,6-bisphosphatase 3 (*Pfkfb3*), cholecystokinin (*Cck*), and insulin-like growth factor binding protein 5 (*Igfbp5*) in the mammary gland were also dynamic in response to treatment. We chose to evaluate Pfkfb3 and Igfbp5 because they are key enzymes in the regulation of glucose (*Pfkfb3* through gluconeogenesis and *Igfbp5* through the growth hormone, insulin-like growth-factor 1 system) at the level of the mammary gland. *Cck* is implicated in satiety, and may therefore be dysregulated in an obesity model. Expression of *Pfkfb3* was affected by both diet (*P* = 0.04) and genotype (*P* = 0.004), but not by the interaction (*P*>0.05). WT HFD dams had numerically higher expression of Pfkfb3 than WT LFD dams (*P*>0.05), and within all HFD dams, expression of *Pfkfb3* was lower in *Tph1*^*-/-*^ than WT dams (*P*<0.05). *Tph1*^*-/-*^ LFD dams also had numerically reduced mammary gland expression of *Pfkfb3* than WT LFD dams (Fig 4C). Diet (*P* = 0.02), genotype (*P* = 0.04), and the interaction (*P* = 0.005) all had an effect on mammary expression of *Igfbp5*. Within WT dams, WT HFD had greater expression than WT LFD (*P*<0.01). This effect was attenuated by *Tph1* deficiency, as *Tph1*^*-/-*^ HFD dams had lower expression of *Igfbp5* than WT HFD dams (*P*<0.01). *Tph1* deficiency alone did not have an effect on mammary expression of *Igfbp5* (*P*>0.05; Fig 4D). mRNA expression of *Cck* in the mammary gland was overall only affected by genotype (*P* = 0.03). Diet alone did not have an effect, as WT HFD and WT LFD dams did not have differential expression of Cck (*P*>0.05). However, within HFD dams, those deficient for *Tph1* had greater expression than WT HFD dams (*P*<0.05). There was no difference in expression between WT LFD dams and *Tph1*^*-/-*^ LFD dams (*P*>0.05; Fig 4E).

## Discussion

Delayed onset of lactogenesis stage II is a significant barrier to successful initiation of breastfeeding. Women who take longer than 72 hours to initiate copious milk secretion are at a greater risk of shorter breastfeeding duration [7]. Numerous studies have correlated DOL with overweight or obesity in women [4,5,6,33]. Breastfeeding offers numerous protective effects, including decreasing the risk of the infant being overweight or obese, leading one researcher to state that “obese women and their offspring who are the most likely to benefit from a longer duration of breastfeeding, are the least likely to do so” [7]. In this study, we have demonstrated the involvement of serotonin in mediating the mammary gland response to HFD. Specifically, we have shown that a genetic deficiency for the rate-limiting enzyme in non-neuronal serotonin synthesis (*Tph1*^*-/-*^) improves the HFD phenotype that may otherwise prevent the successful onset of lactation.

In order to establish obesity in our mouse model, we fed a diet containing 60% kcal% fat throughout pregnancy and lactation. We have previously used this diet, as well as our chosen control diet, to demonstrate the effects of high-fat diet feeding on the ability of the mammary gland to produce milk at the onset of lactogenesis [22]. Previous studies have established different mammary gland phenotypes in HFD dams based on whether they were lean or obese. Obese HFD dams have more and larger adipocytes in their mammary glands compared to lean HFD dams, potentially affecting mammary epithelial cell differentiation and milk yield [34]. Interestingly, dams on the HFD only weighed more than their LFD counterparts on L0, with their body weights leveling out by L10. That HFD dams did not weigh more on L10 is possibly due to the elevated energy expenditures of lactation, in which extra energy is dedicated towards milk production. Obese animals are known to deliver fewer pups and have lower pup survival [33]. Although WT HFD dams only had a numerically higher number of pregnancies to a successful lactation, WT HFD dams dramatically failed to support their pups on day 1 of lactation, as indicated by their negative milk yield. As a consequence, pup mortality was significantly elevated in the WT HFD group. Deficiency of *Tph1* mitigated this phenotype, with *Tph1*^*-/-*^ HFD dams producing the same amount of milk as both of the LFD groups, without any deleterious effects on pup mortality. This finding is of significant clinical value, highlighting serotonin as an important potential pharmacological target to support milk synthesis at the onset of lactation in obese women.

Rodent studies have demonstrated that obesity during lactation results in reduced development and / or differentiation of the alveoli, as opposed to impaired growth or proliferation of the mammary tissue [35]. Histological evaluation of the mammary glands in our study supports this finding, as WT HFD dams had more adipose tissue in their glands, with fewer milk droplets apparent within the luminal space of the alveoli. It is important to note that these glands were taken on L10, when milk yield was similar in all groups. As such, it appears that the WT HFD dams were able to sustain secretory capacity once they had established the lactation, despite the lactation being delayed by at least one day. Notably, both HFD groups had smaller alveoli than their LFD counterparts, and *Tph1*^*-/-*^ dams had more alveoli than WT dams, regardless of diet. Serotonin can have a variety of effects on mammary gland alveolar cell proliferation and differentiation, dependent on both dose and timing of lactation [36]. Given the time sensitivity of this local regulator, further exploration of lactating mammary glands on day 1 is necessary to fully examine the role of serotonin in milk synthesis with respect to DOL and obesity.

Forced weaning of rodents at 10 days of lactation has been shown to trigger epithelial cell death in the mammary gland within hours, characterized by irreversible remodeling of the mammary tissue by proteases and macrophages [37]. In this sense, obesity has been implicated in provoking a “precocious involution phenotype” in the lactating mammary gland. In dams fed a HFD, infiltrating macrophages are elevated in the mammary gland [38]. Serum and mammary gland mRNA expression levels of *Tnfa* are increased during pregnancy and lactation in HFD compared to standard chow dams [38,39,40]. In this study, mammary gland expression of *Tnfa* was shown to be elevated in the WT HFD dams, and this expression was attenuated in the *Tph1* deficient dams. Although protein concentrations of TNFa were not significantly elevated in the WT HFD mammary glands, they were numerically elevated. It is of note that the mammary glands were acquired on L10, and not on the first day of lactation when a more robust response might be expected, based on the milk yield and pup mortality data. Similar to TNFa, elevated levels of CXCL5 in serum have been observed in obese mouse models, and CXCL5 is associated with the onset of obesity and hyperglycemia in serum panel measurements. CXCL5 is stimulated in response to inflammation, and mediates chemotaxis of angiogenic neutrophils [41]. Supporting our hypothesis that 5-HT is involved in mediating an immune-response in HFD mammary glands, *Cxcl5* mRNA expression was elevated in WT HFD mouse mammary glands, but not in *Tph1*^*-/-*^ HFD glands. Finally, *Nos2* mRNA expression was elevated in WT HFD mammary glands relative to both WT LFD and *Tph1*^*-/-*^ HFD groups. In response to inflammatory stimuli, NOS is induced and sustains high levels of nitric oxide that predominate during inflammatory states, such as obesity [42]. Additionally, nitric oxide is necessary for triggering mammary gland involution, as *Nos2*-null mice experience delayed apoptosis of the epithelial cells and extracellular matrix remodeling is decreased [43]. Interestingly, *Nos2* expression was also elevated in *Tph1*^*-/-*^ LFD, perhaps implicating serotonin in the regulation of nitric oxide production, dependent on diet.

In addition to its apparent role in regulation of immune and inflammatory status in the mammary gland, global ablation of *Tph1*^*-/-*^ affected fatty acid dynamics in the lactating mammary gland. Maternal obesity is known to alter milk fatty acid composition, along with decreasing water and carbohydrate content in milk and increasing fat [44]. De novo fatty acid synthesis has been shown to be impaired by administration of a HFD in the mouse mammary gland through inhibition of acetyl-CoA carboxylase [34]. Additionally, fatty acid profiles in milk may play a role in fetal programming [45], with increased fat deposition evident in offspring consuming fatty milk [46] and complications with energy balance and fuel utilization noted in suckling pups of obese dams [47]. Consumption of HFD in rodents before and during pregnancy has been shown to produce obesity in the adult offspring of that pregnancy [48]. While this study did not examine the pups of obese offspring, the milk fatty acid profiles are suggestive of differential transfer of nutrients through the milk to the pups based on genotype and diet. Milk that was acquired within the first few days of lactation demonstrated a fatty acid profile that was responsive to *Tph1* deficiency. Interestingly, abundance of de novo-synthesized lauric acid (12:0) was reduced in *Tph1*^*-/-*^ HFD dams relative to WT HFD dams, while diet-derived oleic acid (18:1c9) was more abundant in *Tph1*^*-/-*^HFD milk relative to WT HFD milk. By L10, de novo fatty acid synthesis was dramatically decreased in both WT and *Tph1*^*-/-*^ HFD mammary glands, as indicated by the reduced abundance of all fatty acids less than 16:0 in length. Conversely, the dietary-derived oleic acid (18:1c9) and linoleic acid (18:2n-6) were increased in abundance in both HFD groups milk on L10. Deficiency of *Tph1*^*-/-*^ did not produce dramatic results in milk composition on L10, potentially corresponding with the improved milk yield in WT HFD dams, both of which demonstrate the glands’ ability to adapt in order to promote pup survival. Nonetheless, given the impact of milk composition on the growth of the infant, and potentially further throughout development with impacts on fetal programming, the effects of serotonin on milk fatty acid composition should be further explored.

The milk fatty acid profiles may be in part explained by the activity of energy-regulating genes in the mammary gland. 6-phosphofructo-2-kinase/fructose-2,6-bisphosphatase 3 (*Pfkfb3*) is known to promote glycolysis through activation of fructose-2,6-bisphosphatase and subsequent activation of phosphofructokinase 1, a key regulatory enzyme in glycolysis. Expression of *Pfkfb3* is increased in WT HFD mouse mammary glands relative to all other groups, suggesting a high nutrient state and subsequent increased glycolytic activity in this group. Deficiency of *Tph1* appears to diminish this effect, decreasing expression of *Pfkfb3* relative to WT dams. Similarly, *Igfbp5* mRNA expression is elevated in WT HFD mammary glands, but not in *Tph1*^*-/-*^ dams. Insulin-like growth factor binding protein 5 is synthesized locally in the mammary gland and has shown to be upregulated at the onset of involution [49]. Additionally, it regulates bioavailability of liver- and mammary-derived insulin-like growth factor 1 [50], which has been demonstrated to protect against apoptosis during involution [51]. In accordance with our immune genes that suggest an involution-like phenotype, *Igfbp5* expression is elevated in WT HFD dams, with *Tph1*^*-*^ deficient dams able to attenuate this response when fed a HFD. Finally, *Cck* expression was elevated in *Tph1*^*-/-*^ HFD dams relative to WT HFD dams. This is of particular interest with respect to fetal programming, as *Cck* is known to serve as an appetite suppressant. Although *Cck* has only been detected in very minute amounts in human breast milk [52], our data would tentatively suggest that perhaps *Tph1* deficiency positively regulates fetal programming of appetite in pups exposed to milk from obese mothers.

It was recently shown that pups suckling from dams administered monosodium glutamate (MSG), which is known to induce obesity, throughout pregnancy were smaller than control pups. Pups that were exposed to MSG during pregnancy and subsequently cross-fostered to a control dam during lactation grew normally. Therefore, the lack of weight gain in pups exposed to MSG during lactation was attributed to reduced milk yield. Indeed, dams exposed to MSG only during pregnancy exhibited less alveoli with altered morphology [53]. Rat pups exposed to MSG during pregnancy and cross fostered to control dams during lactation had normalized body weight, food intake, leptin signaling, lipid profiles, and regulation of insulin compared to rat pups exposed to MSG during both pregnancy and lactation [54]. As such, nutritional manipulation during pregnancy alone is enough to induce alterations in mammary gland morphology. Future studies should evaluate the contribution of serotonin to mammary gland function considering lactation and pregnancy as independent time points.

It is important to acknowledge that genetic interference with serotonin synthesis is likely to have wide-ranging effects. As we chose to use a mouse that was globally deficient for *Tph1*, we cannot rule out that changes in metabolism in the *Tph1*^*-/-*^ mice were not due to metabolic adaptations in other tissues. For example, Crane and coauthors showed in 2014 that *Tph1* deficient mice have enhanced brown adipose tissue (BAT) thermogenesis and are resistant to obesity [20]. Increased BAT thermogenesis is likely to have an overall impact on metabolic homeostasis, including lipid deposition and fatty acid composition of the milk. In our *Tph1*^*-/-*^ mice, therefore, it is possible that improvement of mammary gland function and reduced fat deposition in the mammary gland may be an indirect result of various metabolic changes associated with *Tph1* deficiency.

Our conclusions are limited by several factors that, while representative of lactation physiology, are essential to acknowledge in a controlled animal study. Dams from both treatments fed a HFD required a numerically greater number of pregnancies to achieve a successful lactation through L10, leading to an average greater number of days from initial breeding to birth of a litter that survived through L10. Multiparous dams are more likely to care appropriately for their offspring [55] and, as such, have higher pup survival rates than primiparous dams [56]. As such, gravidity may have had an effect on pup mortality in this study and further studies examining lactation success and HFD should control for gravidity. Additionally, WT HFD dams were unable to support their pups at the beginning of the lactation, likely causing greater pup mortality and making standardization of litter size very difficult. Parameters such as total bone mobilized, and therefore concentrations of calcium in the milk, are affected by the number of offspring [57]. Pups from smaller litters also demonstrated greater weight gain and adiposity when compared to pups from normal-sized litters, suggesting long-lasting effects of over-nutrition as a result of litter size during lactation [58]. Similarly, sex ratio of the pups can have an effect on subsequent postnatal development, manifesting meaningful, but fundamentally different, endocrine and metabolic dysregulations dependent on the sex of the suckling pup [59]. As such, milk composition should be more robustly measured in further obesity studies in which offspring can be normalized and sex ratio balanced.

In conclusion, we have identified the serotonergic system as a potential regulator of the obesity-induced involution phenotype in lactating dams. In our WT HFD dams, there was increased immune and inflammatory expression, along with dysregulated energy and fatty acid activity, at the level of the mRNA. Additionally, WT HFD dams had a reduced ability to feed their pups at the onset of lactation, resulting in a high pup mortality. Reduction of peripheral serotonin via genetic deficiency of its rate-limiting enzyme *Tph1* mediated many of the negative effects of HFD feeding. In obese women who are struggling to initiate lactation, serotonin may be an important pharmacological or molecular target to promote successful breastfeeding. Future research should consider serotonin as an important mediator in the onset of obesity-induced DOL.
